# Adjuvant radiotherapy for the primary treatment of adrenocortical carcinoma: are we offering the best?

**DOI:** 10.1590/S1677-5538.IBJU.2017.0095

**Published:** 2017

**Authors:** Victor Srougi, Jose Bessa, Fabio Y. Tanno, Amanda M. Ferreira, Ana O. Hoff, João E. Bezerra, Cristiane M. Almeida, Madson Q. Almeida, Berenice B. Mendonça, William C. Nahas, Jose L. Chambô, Miguel Srougi, Maria C. B. V. Fragoso

**Affiliations:** 1 Divisão de Urologia, Faculdade de Medicina da Universidade de São Paulo, São Paulo, Brasil; 2 Divisão de Urologia, Universidade de Feira de Santana, BA, Brasil; 3 Unidade de Suprerrenal da Divisão de Endocrinologia da Faculdade de Medicina da Universidade de São Paulo, Brasil; 4 Divisão de Endocrinologia do Instituto do Câncer do Estado de São Paulo, Faculdade de Medicina da Universidade de São Paulo, São Paulo, Brasil; 5 Divisão de Radioterapia, Faculdade de Medicina da Universidade de São Paulo, São Paulo, Brasil

**Keywords:** Adrenocortical Carcinoma, Radiotherapy, Adjuvant, Therapeutics

## Abstract

**Purpose:**

To evaluate the role of ARDT after surgical resection of ACC.

**Materials and Methods:**

Records of patients from our institutional ACC database were retrospectively assessed. A paired comparison analysis was used to evaluate the oncological outcomes between patients treated with surgery followed by ARDT or surgery only (control). The endpoints were LRFS, RFS, and OS. A systematic review of the literature and meta-analysis was also performed to evaluate local recurrence of ACC when ARDT was used.

**Results:**

Ten patients were included in each Group. The median follow-up times were 32 months and 35 months for the ARDT and control Groups, respectively. The results for LRFS (p=0.11), RFS (p=0.92), and OS (p=0.47) were similar among subsets. The mean time to present with local recurrence was significantly longer in the ARDT group compared with the control Group (419±206 days vs. 181±86 days, respectively; p=0.03). ARDT was well tolerated by the patients; there were no reports of late toxicity. The meta-analysis, which included four retrospective series, revealed that ARDT had a protective effect on LRFS (HR=0.4; CI=0.17-0.94).

**Conclusions:**

ARDT may reduce the chance and prolong the time to ACC local recurrence. However, there were no benefits for disease recurrence control or overall survival for patients who underwent this complementary therapy.

## INTRODUCTION

ACC is a rare and lethal disease. One in 1 million individuals will develop this tumor that have a median overall survival time of 1.7 years (1, 2). Most patients are diagnosed when advanced disease is present (3). While 37% present with a localized tumor, 17% present with regional involvement, and 46% with metastatic disease; the 5-year survival probabilities for these disease stages are 62%, 39%, and 7%, respectively (2). Patients who undergo surgical treatment with curative intent have high risk of tumor recurrence of up to 80% (4–7). Because of its aggressive behavior, ACC has substantial clinical importance and elicits continuous medical debate.

The treatment of ACC is challenging and usually involves a multimodal therapy, including systemic cytotoxic therapy and ARDT. Robust scientific evidence that supports the use of ARDT after surgery for the primary treatment of ACC is lacking. Only retrospective studies have been performed to date. These studies include only small cohorts of patients, probably due to the low prevalence of the disease.

The results of early case series studies indicate that ACC is resistant to radiotherapy (8–13). Concomitant with the adoption of mitotane and improvement in radiation techniques, it was later reported that ARDT can improve local control and reduce the chance of tumor recurrence (14, 15). However, the largest study of the primary treatment of ACC found that there were no benefits for patients who received ARDT (16). Taken together, the limitations of retrospective small series and the controversial outcomes observed, indicate that the role of radiotherapy in the adjuvant setting should be further studied.

We performed a systematic review and meta-analysis that aimed to evaluate the use of ARDT after surgery, for the control of local recurrences in the primary treatment of ACC. We also report our clinical experience with ACC by presenting the results of a paired comparison between patients who were treated using surgery followed by ARDT or surgery alone.

## MATERIALS AND METHODS

### Institutional paired match comparison

We performed a retrospective search of our adrenocortical cancer database. We selected data from adult patients who were treated using surgery, between 1 January 1994 and 1 January 2014. The patients were separated into two Groups for analysis based on treatment strategy. The treatment Group consisted of individuals treated with surgery followed by ARDT; the control Group consisted of individuals treated with surgery only. The inclusion criteria comprised reported surgical margins, ARDT performed at our institution, and adjuvant use of mitotane. Mitotane data were not included in the analysis. The periods of use and doses given were inconsistent among patients because of variation in tolerance and toxicity. The exclusion criteria comprised patients that had ARDT for the treatment of recurrence or of secondary lesions, intra-operative rupture of the tumor capsule, and positive macroscopic surgical margins. The required minimal follow-up periods were 2 years after radiotherapy in the ARDT group and 2 years after surgery in the control Group, except for the patients that died before. The Groups were paired for comparison based on surgical margin status and clinical stage according to European Network for the Study of Adrenal Tumors classification guidelines. The Weiss scores among the corresponding pairs never exceeded more than two units. Conformal three-dimensional radiotherapy was applied at the tumor bed for all patients in the ARDT group, within 6 months after surgery. Radiation extension and dose were chosen according to adrenocortical cancer severity (based on margin status, size, stage, and Weiss score). Radiotherapy toxicity was classified according to Radiation Therapy Oncology Group criteria (17).

The Groups were compared using local recurrence-free survival LRFS, RFS and OS outcome variables. Local and distant recurrences were diagnosed using computed tomography magnetic resonance or positron-emitting tomography imaging. The times to local and distant recurrence were considered the periods from the surgery date to the date of the imaging examination that revealed the recurrence. If recurrence did not occur, patients were censored at the date of death or at the date of the last follow-up examination. One patient with single metastatic disease was included in each Group. Both were excluded from the recurrence-free survival analysis. OS time was measured from the surgery date to the date of death. Patients still living at the last date of follow-up were censored in the analysis.

The statistical analysis was performed using Stata^®^ 13.0 (Statacorp, College Station, TX, USA). The Groups were compared using the paired t test for continuous variables and Fisher's exact test for categorical variables. Unpaired t tests were used to compare time to event data. The Kaplan-Meier method was used for the survival analysis (GraphPad Prism^®^ version 6.02 for Windows application). The between-Group differences in end-points were compared using the log-rank test. All tests were 2-sided. A p-value <0.05 was considered to indicate a statistically significant result.

### Meta-analysis for local recurrence

A systematic review of the Medline, Cochrane, and Scopus databases was used to search for English language articles that included adrenocortical carcinoma and radiotherapy, in October 2016. The key words used were adrenocortical cancer, adrenocortical neoplasia, adrenocortical carcinoma, radiotherapy, and radiation (Medline search example: “adrenocortical” AND (“carcinoma” OR “cancer” OR “neoplasia”) AND (“radiotherapy” OR “radiation”)). There was no time limit on publication date. The data were extracted and analyzed by two individuals (V.S. and J.B.). We selected studies of the primary treatment of ACC that reported a paired comparison analysis of the outcomes of surgery alone and surgery followed by ARDT. Articles that included the use of radiotherapy as the initial primary treatment for adrenocortical cancer, or for metastasis, were excluded. To minimize the risk of assessment bias, the NOS was used to qualify the selected studies (18). A score ≥7 was used to indicate a high risk of bias, moderate risk was indicated by a score of 4-6, and a low risk was indicated by a score ≤3. An adequate follow-up length was determined to be ≥2 years. A fixed model was used to estimate the HRs for local recurrence, with 95% confidence intervals CIs. Heterogeneity was quantified using the I^2^ test. The analysis was performed using the Comprehensive MetaAnalysis^®^ version 2.2.064 application (Biostat, Englewood, NJ, USA).

## RESULTS

### Institutional paired match comparison

We found 61 patients who underwent surgery as the primary treatment for adrenocortical cancer and who were being followed at our institution. Twelve of these patients met the inclusion criteria for the ARDT Group and 26 met the criteria for the control Group. After the matched pairing process was completed, there were 10 patients in each Group. There were no significant between-group differences in age, gender, tumor laterality, tumor size, or Weiss score. The results for the patient's demographic characteristics are presented in Table-1. The median follow-up times for the ARDT and control Groups were 32 months (range: 17-42 months) and 35 months (range: 11-198 months), respectively. Local recurrence was diagnosed in 4 of the ARDT Group, and in 6 of the control Group, patients (p=0.19). Distant recurrence occurred in 8 of the ARDT Group, and 7 of the control Group, patients (9 patients per Group; p=1.0). Seven patients died in each Group (p=1.0).

**Table 1:** Patient demographics.

		ARDT group	Control group	*p*
Age (years)*		40 (24-81)	38 (22-63)	0.53
**Gender (%)**				**0.40**
	Male	4 (40%)	1 (10%)	
	Female	6 (60%)	9 (90%)	
**Side (%)**				**1.0**
	Right	3 (30%)	9 (90%)	
	Left	7 (70%)	1 (10%)	
Tumor size (cm)*		12.7 (6–17)	10 (4–15)	0.28
Weiss score*		8 (4–9)	7 (3–8)	0.08

* Median (range)

**ARDT** = Adjuvant radiotherapy

ARDT was well tolerated by most of the patients in this cohort. Grade 1, 2, and 3 toxicity occurred in 2 (20%), 4 (40%), and 4 (40%) patients, respectively. No evidence of late radiation toxicity was reported. The median radiation dose was 54 Gys (range=45-54 Gys). The median time to ARDT was 100 days after surgery (range=27-158 days).

**Figure 1:**
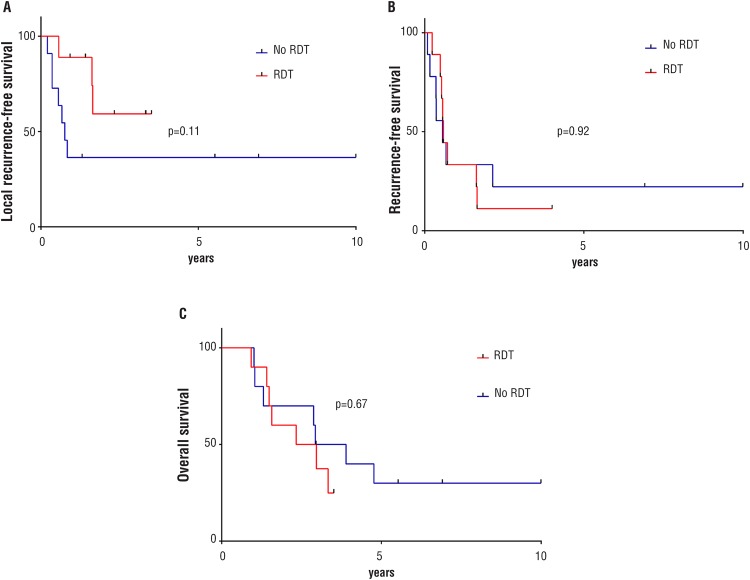
Kaplan-Meier analysis of local recurrence-free survival (A), recurrence-free survival (B), and overall survival (C).

The LRFS times were similar among Groups (p=0.11). The 5-year RFS (p=0.92) and OS (p=0.47) times were also equivalent in both subsets of patients (Figure-1). The mean time for local recurrence was significantly greater in the ARDT Group (p=0.03). The mean times for disease recurrence (p=0.58) and death (p=0.55) were similar in the ARDT and control Groups (Table-2).

**Table 2:** Treatment outcome and time to event results.

		ARDT group	Control group	*P*
Follow-up (months)*		32 (11-43)	35 (11-195)	0.16
**Local recurrence**				
	Number of patients (%)	4 (40)	6 (60)	0.19
	Average time (days)	419 ± 206	181 ± 86	0.03
**Disease recurrence** **				
	Number of patients (%)	8 (89%)	7 (78%)	1.0
	Average time (days)	291 ± 194	225 ± 255	0.58
**Death**				
	Number of patients (%)	7 (70%)	7 (70%)	1.0
	Average time (days)	730 ± 326	929 ± 541	0.42

* Median (range)

** Nine patients analyzed in each group

**ARDT** = Adjuvant radiotherapy

### Meta-analysis for local recurrence

**Figure 2:**
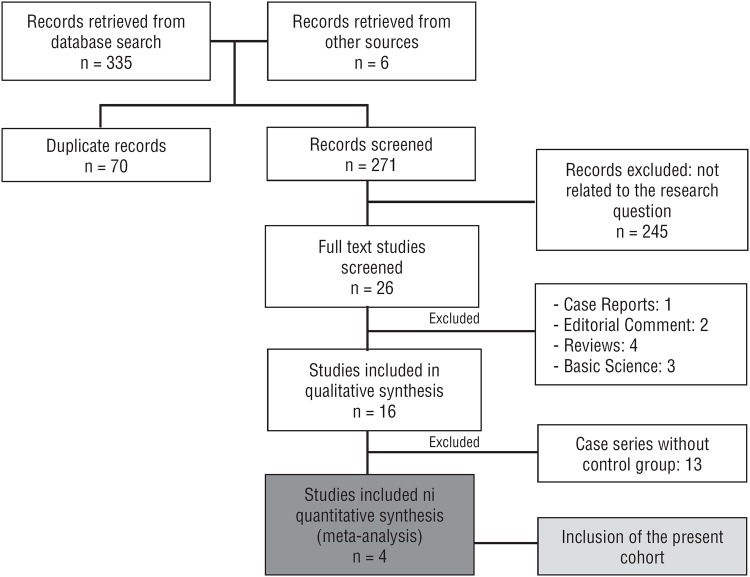
Systematic review flowchart.

**Figure 3:**
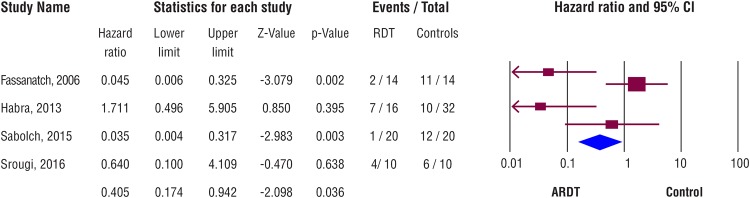
Results of meta-analysis for local recurrence-free survival after ARDT for adrenocortical carcinoma (ACC).

The database search revealed 335 articles; 3 of these articles met the selection criteria (Figure-2). The data from the present cohort were also included in the meta-analysis. All selected articles included results from matched paired series comparing patients who had surgery as the primary treatment for adrenocortical carcinoma, with or without ARDT. Results from a total of 136 patients were included. The NOS indicated that all articles had a low risk of bias (Table-3). The Sabolch et al. article had the lowest score; this cohort included patients who underwent radiotherapy for surgically treated local recurrence (7). A meta-analysis was performed to evaluate LRFS (Figure-3). RFS and OS were not analyzed due to the scarcity of data. Patients who had ARDT had a lower chance of local tumor recurrence, compared with the control Group patients (HR=0.4; CI=0.17-0.94; p=0.036; I^2^=0.79). The outcomes of the selected articles were considered with substantial heterogeneity.

**Table 3:** Newcastle-Ottawa Scale assessment for selected cohort studies.

Articles	Selection				Comparability	Outcome			Score
	Representativeness of the present cohort	Selection of non-exposed	Ascertainment of exposure	Outcome not present at start		Assessment of outcome	Adequate follow-up length	Adequate follow-up	
Fassanatch, 2006	**⋆**	**⋆**	**⋆**	**⋆**	**⋆ ⋆**	**⋆**		**⋆**	8
Habra, 2013	**⋆**	**⋆**	**⋆**	**⋆**	**⋆ ⋆**	**⋆**		**⋆**	8
Sabolch, 2015		**⋆**	**⋆**	**⋆**	**⋆ ⋆**	**⋆**		**⋆**	7
Srougi, 2017	**⋆**	**⋆**	**⋆**	**⋆**	**⋆ ⋆**	**⋆**	**⋆**	**⋆**	9

## DISCUSSION

The results for our study cohort showed that there were no benefits from ARDT for local recurrence control of ACC. However, the analysis of published results indicated that the use of this complementary therapy after surgery for the local control of the disease has some benefit. The survival analysis revealed that ARDT did not affect disease recurrence or OS, which was consistent with previously published results.

The pairing criteria among the selected articles included tumor margin status and ACC stage, which are known major risk factors for patient prognosis (3, 19).

Pathological characteristics that are as important as these criteria were not used in any of the series, except by Sabolch et al., who used tumor grade for pairing (15). We aimed to reduce the effects of this difference by minimizing the difference in the Weiss criteria between pairs, which also affects tumor recurrence (20).

Some factors may have changed the results of radiotherapy for ACC treatment over the years. Mitotane has been widely adopted for adjuvant treatment of ACC, and its use is associated with improved survival (7). In experimental studies, Cerquetti et al. found that mitotane is also a sensitizer for radiotherapy and, therefore, can improve ARDT results (21, 22). Recent case series have comprised patients using mitotane, including most of the articles selected for the meta-analysis. Another important factor that has contributed to the improvement in ARDT outcomes is progress in the development of radiation technology. Three-dimension conformal planning reduces the side effects and enhances the efficacy of external beam radiotherapy. This change has been crucial, considering that the actual recommendation is to irradiate a wide territory because up to 25-30% of the patients may have inter-aortocaval lymph node metastases (2). Consistent with our study cohort results, the analysis of the selected series revealed well-tolerated radiation toxicity; only two cases of late toxicity were reported (14, 16).

The use of ARDT for control of local recurrence is still controversial. Habra et al. found a local recurrence-free rate of 53% for patients who received ARDT, and 67% for a non-ARDT Group; the between-group difference was not statistically significant (16). This finding motivated our study, which shows similar results. Of noteworthy, patients who underwent ARDT did have a longer mean time to development of local recurrence, which was approximately 240 days more than the patients in the control Group. The use of a longer follow-up time could reveal that the chances of local recurrence are the same for patients receiving or not receiving ARDT. However, this finding may justify the use of ARDT for ACC treatment since an incremental increase in the time interval for local disease control could improve quality of life for patients with this aggressive neoplasm.

The results of our meta-analysis suggest that the use of ARDT might increase LRFS. Nonetheless, a definitive treatment paradigm cannot be developed from these results because there was substantial heterogeneity in the outcomes in the published series. Furthermore, it should be emphasized that all evidence to date also indicates that ARDT does not affect disease recurrence or OS (14, 16). It is reasonable to question whether ARDT should be used if it does not improve survival.

This study was limited by its retrospective design and small sample sizes. Our meta-analysis included only four paired retrospective case series. However, given the low prevalence of ACC and the limited number of articles available on the use of ARDT, our work represents the highest level of available evidence. We attempted to minimize sample bias via the strict selection of paired studies; the NOS results indicated the presence of a uniform quality among the articles included in the meta-analysis. Nevertheless, only a multi-institutional randomized trial will provide robust evidence for the role of ARDT in the treatment of ACC.

## CONCLUSIONS

ARDT may decrease the chances and delay the time to ACC local recurrence. However, benefits for RFS and OS have not been found. The current evidences lack quality and highlights the need for prospective studies. Considering the rarity of ACC, a joint multi-institutional effort should be implemented to study this problem. Until then, the real advantages of ARDT will remain uncertain.
